# Creating Meiotic Recombination-Regulating DNA Sites by *SpEDIT* in Fission Yeast Reveals Inefficiencies, Target-Site Duplications, and Ectopic Insertions

**DOI:** 10.3390/biom14081016

**Published:** 2024-08-16

**Authors:** Reine U. Protacio, Seth Dixon, Mari K. Davidson, Wayne P. Wahls

**Affiliations:** Department of Biochemistry and Molecular Biology, University of Arkansas for Medical Sciences, Little Rock, AR 72205-7199, USA; rmprotacio@uams.edu (R.U.P.); davidsonmarik@uams.edu (M.K.D.)

**Keywords:** allele replacement, gene targeting, genome editing, homologous recombination, mutagenesis, CRISPR, *SpEDIT*, yeast, *Schizosaccharomyces pombe*

## Abstract

Recombination hotspot-activating DNA sites (e.g., *M26*, *CCAAT*, *Oligo-C*) and their binding proteins (e.g., Atf1-Pcr1 heterodimer; Php2-Php3-Php5 complex, Rst2, Prdm9) regulate the distribution of Spo11 (Rec12)-initiated meiotic recombination. We sought to create 14 different candidate regulatory DNA sites via bp substitutions in the *ade6* gene of *Schizosaccharomyces pombe*. We used a fission yeast-optimized CRISPR-Cas9 system (*SpEDIT*) and 196 bp-long dsDNA templates with centrally located bp substitutions designed to ablate the genomic PAM site, create specific 15 bp-long DNA sequences, and introduce a stop codon. After co-transformation with a plasmid that encoded both the guide RNA and Cas9 enzyme, about one-third of colonies had a phenotype diagnostic for DNA sequence changes at *ade6*. PCR diagnostics and DNA sequencing revealed a diverse collection of alterations at the target locus, including: (A) complete or (B) partial template-directed substitutions; (C) non-homologous end joinings; (D) duplications; (E) bp mutations, and (F) insertions of ectopic DNA. We concluded that *SpEDIT* can be used successfully to generate a diverse collection of DNA sequence elements within a reporter gene of interest. However, its utility is complicated by low efficiency, incomplete template-directed repair events, and undesired alterations to the target locus.

## 1. Introduction

Eukaryotes have a sexual lifecycle with alternating haploid and diploid states that are interconverted by meiosis and fertilization (or conjugation). High rates of correctly positioned homologous recombination (HR) in meiosis promote the faithful transmission of chromosomes between generations [1,2] and create genetic diversity upon which natural selection acts [3,4,5]. In diverse eukaryotic species and taxa, the meiotic recombination events cluster at “hotspots” that control its frequency distribution across the genome [6,7,8]. As is the case for transcription, discrete DNA sites and their binding proteins (e.g., Atf1-Pcr1-*M26* protein–DNA complexes) control locally the rates of meiotic recombination (at hotspots) in the genome (e.g., [9,10] and refs therein).

The precisely regulated positioning of meiotic recombination by DNA site-specific protein-DNA complexes was first discovered using the *ade6* reporter gene in the fission yeast *Schizosaccharomyces pombe* [11,12,13]. Subsequently, the regulatory DNA sites were shown to be active elsewhere in the fission yeast genome [14,15]. So far, five distinct classes of hotspot-activating DNA sites (*M26*, *CCAAT*, *Oligo-C*, *4095* and *4156*) have been defined functionally at single bp resolution in fission yeast [11,13,16,17]. Notably, the recombination-regulating activities of DNA sites and binding proteins discovered in *S. pombe* are conserved functionally in other, highly diverged species [6,18]. Lastly, an insightful, powerful genetic screen in fission yeast, using randomized DNA sequences that were 15 or 30 bp in length, uncovered hundreds of additional, candidate regulatory elements [17]. That screen exploited a high-throughput, qualitative method to score for increased recombination between one allele of the *ade6* gene in the chromosome and a heteroallele of *ade6* in a plasmid. We wanted to test directly whether those short DNA sequences, named “HES” elements, promote meiotic recombination between two homologous chromosomes. In order to do so, we first sought to recreate independently 14 of the candidate regulatory DNA sequence elements, plus one well-matched control allele, within the *ade6* reporter gene.

In fission yeast, reverse genetics via gene targeting (also known as “precise genome editing”) has historically been achieved either by using two successive rounds of gene targeting [19,20] or by a more efficient pop-in, pop-out (PIPO) methodology [21,22]. A related, PCR vectorette-based targeting method is as efficient but is imprecise because it places additional changes into the target locus [23,24]. More recently, several laboratories have developed fission yeast-optimized, CRISPR-Cas9-based genome editing approaches [25,26,27,28,29,30]. One of these approaches, *SpEDIT*, was reported to be exceptionally efficient at placing bp substitutions into the *ade6* coding region (efficiency of 100%, n = 150) [25]. Thus, the objective of this study—to create individually, via bp substitutions, fifteen different clones that each harbor a different DNA sequence at a precise location within the coding region of the *ade6* gene—also provided a “real-world” test of the recently developed, fission yeast-optimized, *SpEDIT* genome editing tool.

## 2. Materials and Methods

### 2.1. DNA Oligonucleotides, Guide RNA, and DNA Templates

The sequences of DNA oligonucleotides (oligos) are listed in Table 1. To express the guide RNA (gRNA) for *ade6*, the complementary oligos Ade6sgRNA-F (F, forward) and Ade6sgRNA-R (R, reverse) were annealed together to generate dsDNA for golden-gate cloning into the pLSB–Kan expression vector, as described [25]. To generate the homologous recombination templates, two 100 nt-long oligonucleotides with partially overlapping complementarity (20 bp at their 3′ ends) were annealed together and extended, as described [31]. First, universal oligo HRuniv-R was annealed in combination with each of the HES-specific oligos (e.g., HES92-F, HES95-F, etc.), as well as with one negative control oligo that encodes the stop codon but does not generate a HES DNA site (HEScon-F); then, high fidelity DNA polymerase was used to create the full-length dsDNA molecule of 180 bp. In the second step, those 180 bp-long dsDNA molecules were used as templates for PCR using oligos ade6+63-F and ade6+258-R as primers to generate the 196 bp-long templates for CRISPR-Cas9-mediated editing (described below). After genome editing, oligos ade6-57-F and ade6+984-R were used for PCR diagnostics and for Sanger DNA sequencing.

### 2.2. Yeast Strains and Culture

All experiments employed wild-type (*h*^−^) yeast strain WSP 3776, which is available upon request. Yeast were cultured using standard methods [32,33], with nitrogen base agar (NBA) or liquid serving as minimal media and yeast extract agar (YEA) or liquid serving as rich media [21,22]. Media were supplemented as necessary with adenine (100 µg/mL) or G418 (100 µg/mL).

### 2.3. Genome Editing and Diagnostics

We used the *SpEDIT* system and published protocols for CRISPR-Cas9-mediated editing [25] of the *ade6* gene. The CRISPR4P program [34] was used to design a gRNA for *ade6* that was positioned near the desired site of editing. Annealed DNA oligos that encode the gRNA were inserted via golden-gate cloning into the expression vector pLSB-Kan, which encodes both the gRNA and Cas9 enzyme [25]. A lithium acetate transformation method [35,36] was used to cotransform the Cas9/gRNA-expressing plasmid (200 ng), one of the 196 bp-long homologous recombination templates (500 ng) and boiled salmon sperm carrier DNA (20 µg) into fission yeast strain WSP 3776. Cells were plated onto YEA that contained G418 and incubated at 32 °C for 4 days to select for transformants; the frequencies of red and white colonies (which are diagnostic for *ade6* mutant and wild-type, respectively) [22,37] were tabulated. Subsequently, red colonies (candidates) were streaked onto non-selective media (YEA without G418) to permit plasmid loss [25]. Where indicated, the *ade6* mutant phenotypes of candidate clones were confirmed by plating each clone in parallel on minimal NBA media that contained or lacked adenine [37]. A smash and grab method [38] was used to prepare genomic DNA from 5 mL of an overnight YEL culture. Genotypes were determined using standard PCR methods and by Sanger DNA sequencing (using oligos ade6-57-F or ade6+984-R as primers).

## 3. Results

### 3.1. Objective, Experimental Design, and Approach

**Figure 1:**
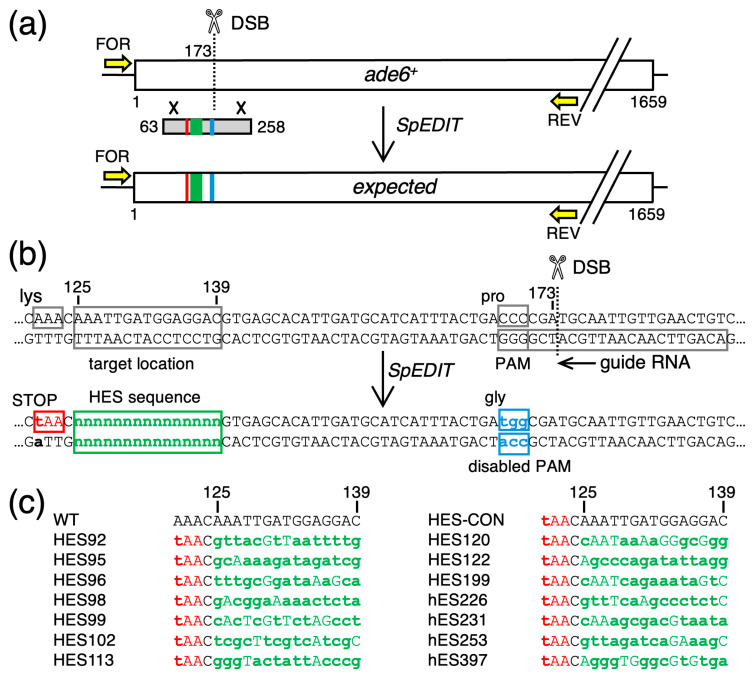
**CRISPR-Based Approach to Generate Candidate Regulatory DNA Sites Within the *ade6* Reporter Gene.** (**a**) Diagram of the *ade6* ORF (*box*) shows the position of the recombination-initiating dsDNA break (*DSB, scissors*), dsDNA template molecule for recombination-mediated repair (*grey box*), features within that template (*colored rectangles*), and recombination events (×) between the genomic DNA and the template DNA. Successful genome editing (*SpEDIT*) transfers the features from the template to the genome. (**b**) As in panel (**a**) but zoomed in to show the relevant DNA sequences and the position of the guide RNA. Base pair substitutions (*bold lowercase*) are used to generate a stop codon (*red*), to create the HES sequence (*green*), and to inactivate PAM (*blue*). The same symbols and color codes are used in subsequent figures. (**c**) Sequences of the HES DNA elements (*green*) and bp substitutions needed to create those DNA sequences; the adjacent stop codon (*red*) provides a marker for analyses of meiotic recombination.

Our objective was to create 15 fission yeast strains that differed from each other only by their DNA sequences between nucleotide positions 125 and 139 of the *ade6* ORF (near the 5′ end) (Figure 1). Fourteen of the strains would each harbor a different, candidate recombination hotspot-activating DNA sequence (HES) [17]; the one other strain, with wild-type DNA sequence in this region, would serve as the control (CON). The HES DNA elements and sequences are color-coded *green* in the figures. All 15 of the strains would also be engineered to contain a stop codon at positions 121–123 (color-coded *red*), which serves two purposes. First, the stop codon would render the modified cells auxotrophic for adenine, thus providing a phenotype (red colony color) to help screen for allele replacements. Second, the stop codon provides a marker for future analyses of meiotic recombination rates. In brief, it allows one to score for Ade^+^ spore colonies from crosses between haploid parents with different alleles of *ade6* (e.g., [39]). Lastly, each of the engineered strains would also have an inactivated protospacer adjacent motif (PAM site) at positions 168–170 (color-coded *blue*); the rationale for including this change is described below. To avoid perturbing the overall structure or spacing of chromosomal elements at the *ade6* locus, we chose to use bp substitutions (rather than insertions or deletions) to create or ablate the DNA sequence features. This approach required designing a total of 12 to 18 bp substitutions per clone (Figure 1b,c). For each clone, all of the designed changes map within a 50 bp-long region of the *ade6* ORF (Figure 1b).

We chose to use the *SpEDIT* methodology to modify the *ade6* gene because this approach is straightforward and has been shown previously to be very efficient at introducing bp substitutions into *ade6* [25]. The CRISPR4P program [34] was used to design the optimal gRNA for our *ade6* target (Figure 1b), which we expressed from pLSB-Kan [25]. We used that one vector, which encodes both the *ade6*-targeting gRNA and a fission yeast codon-optimized Cas9 protein, for all of the allele replacement experiments.

For each of the 15 different allele replacements, we constructed a 196 bp-long, dsDNA template for homologous recombination (HR)-mediated repair of the DSB; that template maps to nucleotide positions 63–258 of the *ade6* ORF (Figure 1a). The 50 bp-long region that contained the desired bp changes was centrally located within the 196 bp-long HR template (Figure 1a,b). The mechanism and desired outcome of the genome editing experiments are shown in Figure 1b. In brief, CRISPR-Cas9 should catalyze the formation of DSBs at *ade6* in the genome; the DNA ends would interact with the dsDNA template; then, repair should copy genetic information from the template to the genome. A repair tract covering the 53 bp to the left of the DSB would be necessary and sufficient to simultaneously modify (edit/replace) DNA sequences at the genomic PAM site (*blue*), at the target location for HES DNA sites (*green*), and at the stop codon (*red*) (Figure 1b). The bp substitutions which inactivate the PAM site were included to prevent the gRNA-loaded Cas9 enzyme from catalyzing multiple cycles of endonucleolytic cleavage at the genomic target after the first round of editing.

### 3.2. SpEDIT Induces Changes in the Length and Sequence of DNA at the ade6 Gene

**Figure 2:**
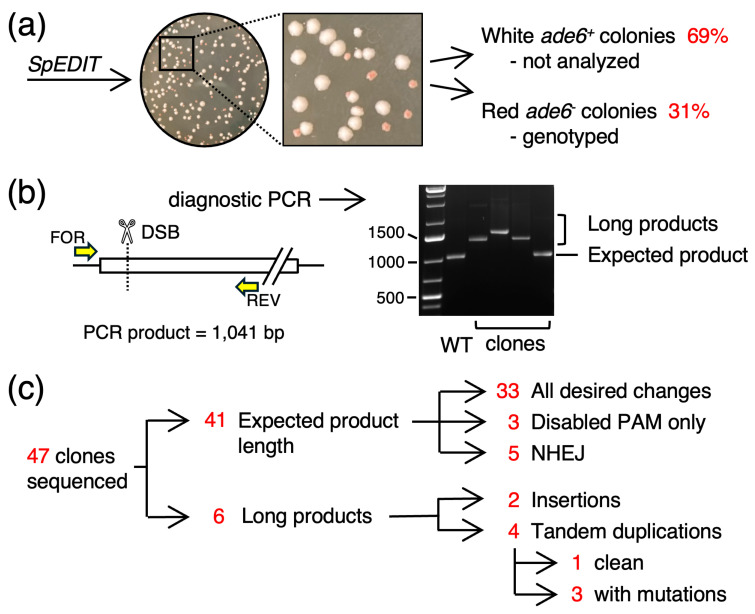
**Efficiency of Targeting at the *ade6* Locus.** (**a**) G418-resistant transformants were screened for red colony color on YEA media, which is diagnostic for mutations within the *ade6* target gene. The structures and DNA sequences of the *ade6* locus from candidate (red) colonies were analyzed. (**b**) Agarose gel shows examples of PCR products that had the expected length and unexpected lengths. (**c**) Summary of results lists each class of alteration detected within the *ade6* gene; data for each class are presented in subsequent figures.

For each allele replacement experiment, we co-transformed wild-type (*ade6^+^*) cells with the *ade6*-targeting, gRNA-Cas9 expressing plasmid, one of the 15 different HR templates, and salmon sperm carrier DNA. We then selected for G418-resistant transformants on YEA media and scored for the red colony color (Figure 2a) that is a phenotype of hypomorphic or null mutations in *ade6* [22,37]. Each of the attempts yielded red colonies (range of 18% to 45% per experiment and per template) and the overall mean frequency of the red colonies was 31% (SD = 8%, n = 6332). This frequency was significantly lower than that reported previously for *SpEDIT*-mediated editing of the *ade6* gene (100% red colonies, n = 150) [25]. The differences in the observed frequencies between the two studies are attributable predominantly to differences in the numbers and distributions of bp substitutions in the template DNA molecules (see **Discussion**).

Clones that were good candidates for successful editing of *ade6* (by virtue of red colony color on YEA media) were cured of the plasmid, streak purified two additional times, and tested for their loss of plasmid by patching them in parallel on media that contained or lacked G418. Then, the genomic DNAs of 47 clones were used as templates for PCR amplification of the *ade6* gene (Figure 2b). Unexpectedly, six of these clones had gene lengths that were substantially larger than wild-type; subsequently, DNA sequencing identified an additional five clones that had changes to DNA sequence length that were too short to be detected by agarose gel electrophoresis of the PCR products (Figure 2c and subsequent figures). We concluded that the *SpEDIT* approach had triggered changes to the *ade6* target gene; however, those insertions and deletions (indels) were aberrant, imprecise outcomes that deviated from the expected, precise genome editing. Additional observed changes to the *ade6* target locus, and how we parsed those changes into discrete classes of events (summarized in Figure 2c), are described in greater detail below.

### 3.3. Successful, Partially Successful, and Unsuccessful Editing of ade6

For each clone, we sequenced both strands of *ade6* DNA. For most clones, aligning the DNA sequence to that of the wild-type *ade6* gene was sufficient to reveal unambiguously the nature of the changes to the *ade6* target locus. For other clones, BLAST searches against the fission yeast reference genome and the non-redundant pan-organism genome database, coupled with manual validation, were required to diagnose the nature of the changes to the *ade6* target locus. These processes allowed us to define multiple different classes of changes to the *ade6* gene (summarized in Figure 2c). We list their properties and specific conclusions here; mechanisms for their genesis are discussed subsequently (see **Discussion**).

**Figure 3:**
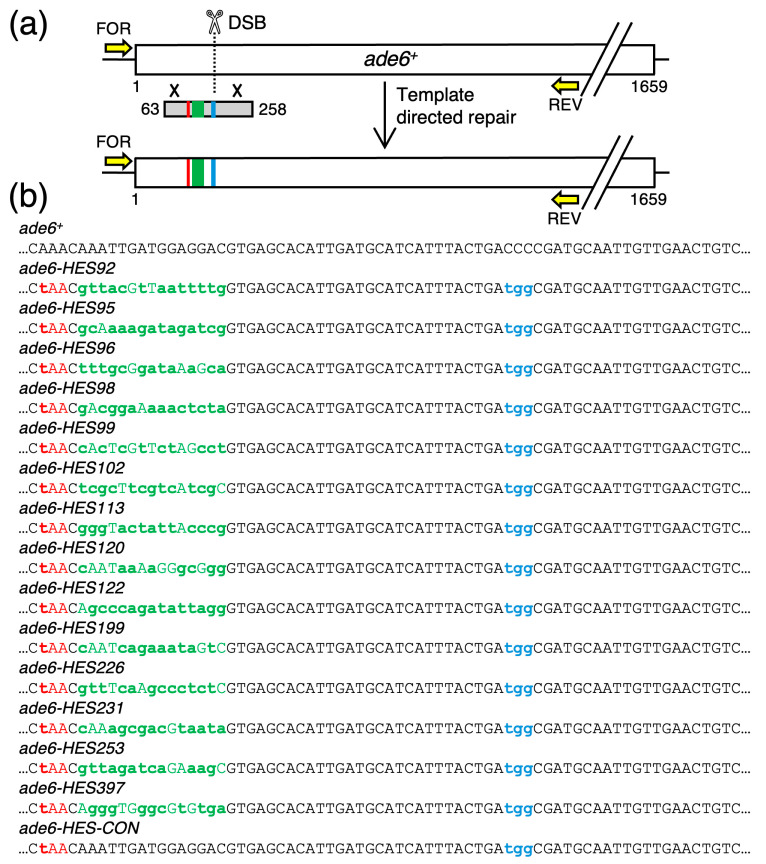
**Complete (Successful) Templated-Directed Modifications of the *ade6* Target Gene.** (**a**) Diagram of approach and results. Correctly positioned recombination events (×) are required to transfer all of the desired elements (*color-coded rectangles*) into the genome. (**b**) The relevant DNA sequences are shown for the parental strain (*ade6^+^*) and for one correctly edited, representative clone for each of the 14 different HES DNA site elements, plus the one control (*CON*).

One class of clones (n = 33), which constituted 22% of all transformants, had correctly generated the stop codon (*red*), introduced the HES DNA sequence (*green*), and mutated the PAM site (*blue*) elements in the *ade6* gene (Figure 3). We concluded that *SpEDIT* can be used successfully to engineer a collection of at least 18 (potentially more) individual bp substitutions distributed over a 50 bp-long region of the target locus. For example, in our case we successfully obtained at least one correct clone each for the 14 different candidate recombination hotspot-activating DNA sequences (HES) and for a well-matched control (CON). The impacts of those DNA sites on meiotic recombination will be the subject of a future study. More generally, this class of clones provides compelling evidence for continuous, complete, template-dependent repair tracks that extend through the region with the bp substitutions.

**Figure 4:**
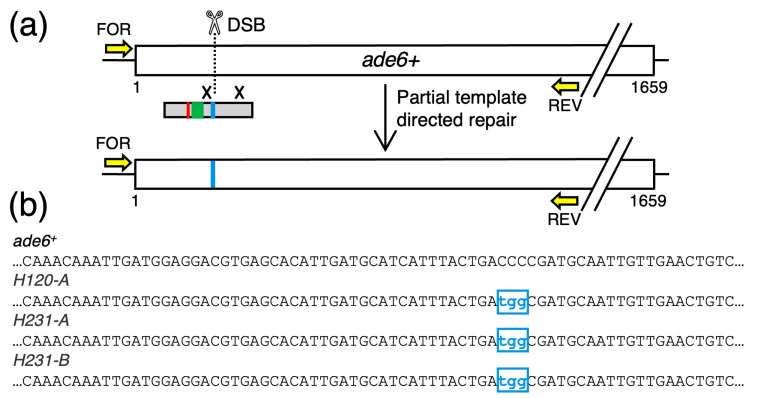
**Incomplete Template-Directed Modifications of the *ade6* Gene.** (**a**) Diagram of approach and results. Some recombination events (×) flanking the DSB transfer only a subset of the desired elements (*color-coded rectangles*) into the genome. (**b**) Representative sequences show correct editing very close to the DSB, at the PAM site (*blue*), but no editing at the more distal bp substitutions (*green* and *red* in panel (**a**)).

A second class of clones (n = 3) had introduced as intended each of the three bp substitutions at the PAM site (*blue*), but had not introduced any of the designed bp substitutions at the position of the HES element (*green*) or the stop codon (*red*) (Figure 4). We concluded that these clones had successfully undergone gRNA-directed DSB formation, interaction of the broken DNA ends with the HR template, and template-directed repair close to (within 4–6 bp of) the DSB. However, the template-directed repair failed to extend to the more distal bp substitutions. In other words, these clones had undergone continuous (of three successive bp) but incomplete (not extending to more distal bp) HR template-dependent repair. Parenthetically, the three bp substitutions that was used to ablate the PAM site is a codon missense mutation that replaces a proline with glycine in the Ade6 protein (Figure 1b). Interestingly, the three independently derived clones (Figure 4b) that passed the colony color screen exhibited a hypomorphic or unstable phenotype in subsequent plating assays, as has been reported for some other missense mutations in *ade6* [22].

**Figure 5:**
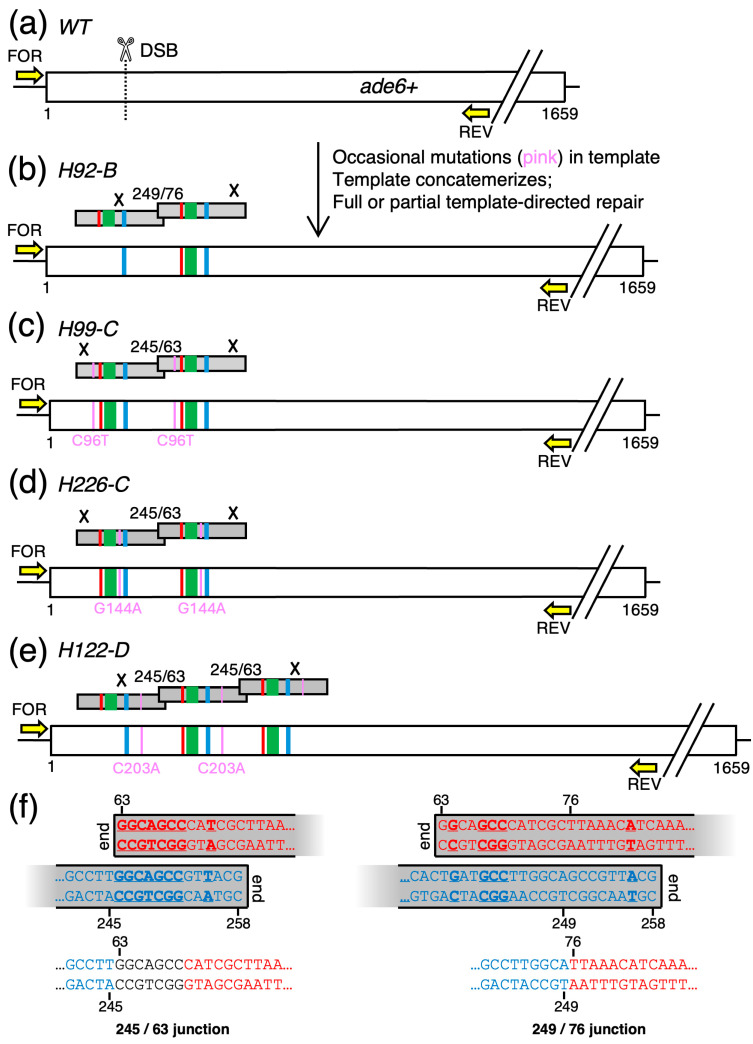
**Template-Directed Duplications of the Targeted DNA Region Within *ade6*.** Diagrams show organization of elements for (**a**) normal-length, wild-type *ade6* and (**b–e**) a subset of clones with increased length. These clones had one or more additional copies of DNA sequence from the template molecule (*grey box*) at the correct position in the target. Some clones (**b**,**e**) had incomplete incorporation of features distal to the DSB (as in Figure 4) and some clones (**c**–**e**) had additional, unplanned bp mutations (*pink*). The diagrams show how the concatemerization of templates prior to repair and the positions of subsequent recombination events (×) produce these types of clones. (**f**) A mechanism for concatemers. Overlapping microhomologies (underlined) near the 5′ and 3′ ends of the linear, dsDNA template molecules (*top*) likely contribute to the formation of the junctions (*bottom*) within the concatemers.

A third class of clones (n = 4) also had clear evidence for template-dependent editing of the *ade6* target (Figure 5). Remarkably, these clones had not simply affected the intended single bp substitutions; they had undergone editing in a way that introduced one or more additional copies of the template DNA into the genome (Figure 5b–e). Notably, all five of the junctions between the tandem repeats occurred near microhomologies located close to the 5′ and 3′ ends of the templates (Figure 5f). These microhomologies likely contributed to concatemerization of template DNA molecules prior to their use for template-directed repair (see **Discussion**).

Interestingly, the template-directed duplications of the targeted DNA region within *ade6* (Figure 5) provided additional evidence for the existence of both continuous, complete repair tracts (as in Figure 3) and continuous but incomplete repair tracts (as in Figure 4). This can be visualized easily by comparing the schematic diagrams of features (color-coded rectangles) in Figure 5: For some clones, all features became edited into the genome (Figure 5c,d); for other clones, only a subset of features were edited into the genome—and in every case the transferred features were all continuous from the location of the DSB up to a distal point, beyond which no features were incorporated (Figure 5b,e).

A subset of the template-directed duplications of the targeted DNA region within *ade6* had another, intriguing feature. These clones harbored unplanned bp substitutions (i.e., mutations) within the template-directed repair tracts (Figure 5c–e). Our current data cannot distinguish whether these stemmed from pre-existing mutations within the template DNA or arose de novo during the course of template-directed repair (see **Discussion**).

**Figure 6:**
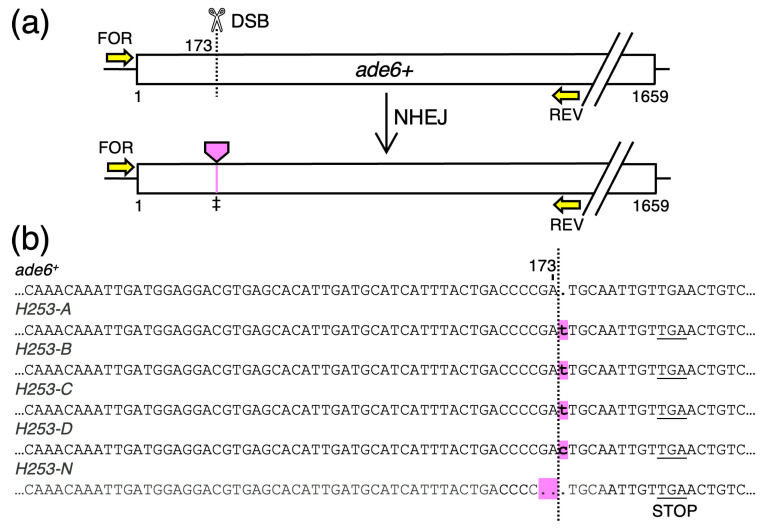
**Template-Independent Modifications of *ade6* by Non-homologous End Joining.** (**a**) Diagram of findings and mechanism. (**b**) DNA sequences of representative clones that had an *ade6* mutant phenotype and mutations (*pink*; ‡ in panel (**a**)) within the *ade6* ORF without incorporating any of the engineered bp substitutions. The short insertions or deletions (*pink*) cluster precisely at the site of the DSB; these indels are diagnostic for non-homologous end joining. The +1 and −2 frameshift mutations within the *ade6* ORF each trigger the use of an out-of-frame nonsense codon (STOP) nearby.

A fourth class of clones (n = 5) had very small deletions (−2 bp) or insertions (+1 bp) located precisely at the site of the recombination-initiating DSB (Figure 6). These mutations are diagnostic for non-homologous end-joining (NHEJ)-mediated repair of the DSB. Interestingly, each of the five tiny indels created a functionally equivalent shift in the reading frame for translation, resulting in each of the five clones using the same stop codon close to the site of the mutations. The termination of protein synthesis near the amino-terminal end of the Ade6 protein explains why these mutants were recovered in the screen. NHEJ can produce other types of changes (e.g., in-frame mutations) that were not recovered by the screen, which has implications for the efficiency with which NHEJ events are detected (see **Discussion**).

**Figure 7:**
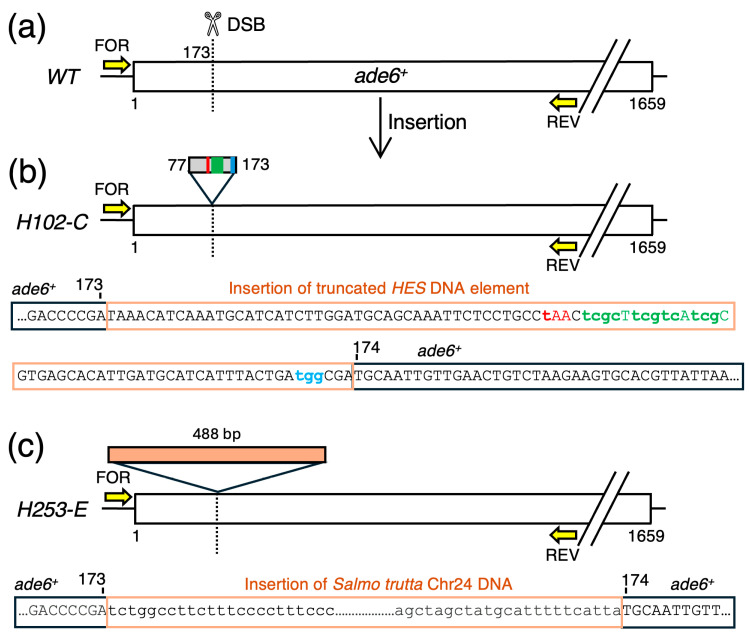
**Insertions of Ectopic DNA within *ade6*.** The diagram shows the organization of the *ade6* gene for a subset of clones with increased length. (**a**) Location of DSB within *ade6* and the non-homologous integrations of (**b**) template DNA sequences or (**c**) salmon sperm carrier DNA. The DNA sequences of the insertion junctions show that the ectopic DNAs integrated precisely at the site of the DSB by a non-homologous end-joining mechanism.

A fifth class of clones (n = 2) had changes that were also diagnostic for NHEJ, but were quite different from the NHEJ events described above. These clones had relatively large segments of DNA inserted directly at the position of the DSB (Figure 7). One of the clones contained an insertion of HR template DNA sequences (Figure 7a) and the other clone had, unexpectedly, the insertion of DNA sequences derived from chromosome 24 of *Salmo trutta* (Figure 7c).

## 4. Discussion

The objective of this study—to create individually via bp substitutions clones that harbor different DNA sequence elements at the identical position within the *ade6* gene—provided a “real-world” test of a recently developed genome editing tool.

### 4.1. Efficiency of Precise Genome Editing by SpEDIT

We tested thoroughly, using 15 different gene targeting templates, the fission yeast-optimized, CRISPR-Cas9-based, *SpEDIT* methodology (Figure 1) [25]. About one-third of transformants had a phenotype diagnostic for DNA sequence changes within the *ade6* target gene (Figure 2) and 22% of transformants had all of the desired bp changes in *ade6* (Figure 3). Overall, within a time span of seven months, we successfully generated and confirmed the integrity of at least one correct clone for each of the 15 gene targeting constructs. Thus, in terms of effort required and likelihood of success, the utility of *SpEDIT* is similar to that of other CRISPER-based editing systems for fission yeast [26,27,28,29,30], to that of precise genome editing via pop-in, pop-out (PIPO) homologous recombination [21,22], and to that of PCR vectorette-based gene targeting [23,24].

### 4.2. Mechanisms for Diverse On-Target Alterations Created by SpEDIT

Although CRISPR-based approaches for genome editing are often touted as being “scarless” [25,34,40,41], our study revealed multiple different classes of undesired alterations to the target locus (Figure 4, Figure 5, Figure 6 and Figure 7). Remarkably, each of the observed outcomes provides insight into the molecular steps required for genome editing via CRISPR—and how things can go wrong.

Early in the process, the gRNA-loaded Cas9 enzyme finds its target in the genome and introduces the recombination-initiating DSB. The homology search step is not rate-limiting in fission yeast [42] and the efficiency of DSB catalysis by the *SpEDIT* system can approach unity, based on the prior report of high-efficiency editing at the *ade6* and *ura4* genes [25]. In contrast, only about one-third (31%) of our transformants had a phenotype diagnostic for DNA sequence changes within *ade6* (Figure 2). Since a given DSB is typically repaired by either HR with the template molecule (which should introduce the template-directed mutations, Figure 1) or by NHEJ (which is mutagenic) [43,44], we can infer that our gRNA–Cas9 complex was inefficient at finding or cutting its target DNA site in the genome. However, it remains possible that the DSBs were induced more efficiently than inferred, but that some of those DSBs were repaired in a way that did not produce an *ade6* mutant phenotype (see next paragraph).

Once DSBs have formed at the target locus, processed DNA ends flanking the DSB engage with the introduced dsDNA template for repair of the DSB via high fidelity, error-free HR (the preferred, default pathway for repair of DSBs) [43,44]. If no template is available, or when the error-free HR pathway fails, cells use error-prone NHEJ to repair the break [43,44]. The observed, very short (1–2 bp), indel-associated mutations located precisely at the site of the DSB (Figure 6) are diagnostic for use of the backup NHEJ pathway. This use of the NHEJ pathway demonstrates successful formation of DSBs but failures of the HR-dependent repair. We note that all of these identified NHEJ events involved a functionally equivalent frameshift (+1 or −2) that led to a stop codon about four codons downstream of the DSB location (Figure 6b). It is possible that other outcomes of NHEJ had occurred (e.g., mutations or junctions that had no frameshift), but failed to produce an *ade6* mutant phenotype. This seems likely because a subset of bp mutations in *ade6* are translationally silent, not all missense mutations inactivate the Ade6 protein, and small DNA indels that do not corrupt the reading frame downstream can yield catalytically active protein [22]. Thus, because we genotyped only clones that had an *ade6* mutant phenotype (Figure 2), our approach might have underestimated the number and frequency of clones with NHEJ.

Failures to engage productively the HR-based pathway induce additional genotoxic insults, as shown directly by the integration of ectopic DNA molecules at the site of the DSB (Figure 7). The fact that we observed non-homologous integrations of both the template DNA (Figure 7a) and salmon sperm carrier DNA (Figure 7b) (which has been reported previously for CRISPR-based editing in fission yeast [45]) suggests that diverse types of ectopic DNAs can be stuffed into the DSB by NHEJ. Given that 29% of the observed NHEJ events had insertions of ectopic DNAs (combined data of Figure 6 and Figure 7), we conclude that this type of on-target, CRISPR-mediated insertional mutagenesis is fairly common. This idea is consistent with (and further supported by) the independent findings of a previous study [45].

If ends of DNA at the genomic DSB engage successfully for repair from the template, and if resolution of the HR events occurs distal to the locations of features (e.g., bp substitutions) within the template, then those features will be incorporated (edited/placed) into the genome, as shown schematically in Figure 1 and documented by data in Figure 3. The fact that we had multiple features within our *ade6* template (inactivated PAM, HES DNA site, stop codon)—which involved up to 18 bp substitutions within a 50 bp-long region (Figure 1b)—allowed us to detect incomplete template-directed repair. Some of these events manifest as template-directed repair (editing) very close to the DSB (at the PAM site) without successful editing of more distal features (Figure 4). (The same type of result, incomplete editing, was also observed when *SpEDIT* was used to introduce bp substitutions in the *ura4* gene [31]). In other cases, the repair track extended to include more distal, but not all, features (e.g., Figure 5b,e). Notably, the repair tracks were all continuous in that they included all of the bp substitutions (features) from the position of the DSB up to a given point, beyond which they contained none of the intended bp substitutions (features). Thus, there is a distance-dependent decay in efficiency of editing relative to the position of the DSB. A model for stochastic positioning of where the HR events are resolved (“×” in the schematic diagrams) can explain fully the data. The distance-dependent effects can also explain why a prior study observed 100% efficiency of editing at *ade6* (all of the designed bp changes were within 4–6 bp of the DSB) [25], whereas only a subset of our edited clones had had full editing at *ade6* (which requires bp changes that span up to 53 bp away from the DSB, Figure 1b). Indeed, if we ignore the two types of clones of the NHEJ class (small indels or large insertions at the site of the DSB), all of the remaining *ade6* mutants that we analyzed had successfully edited the PAM site (within 4–6 bp of the DSB) (see summary of results in Figure 2c).

As mentioned above, some types of mutations introduced by NHEJ (e.g., in-frame, silent, or missense mutations) might fail to create a phenotype that passes the primary colony color screen. A similar logic applies for the incomplete template-directed repair events, such as for the missense substitutions introduced at the PAM site (Figure 4). In other words, our primary screen might have failed to detect a subset of clones with either template-directed (albeit incomplete) repair or NHEJ events. We note that we have no direct experimental evidence that either postulate is true and that either outcome would constitute a “failure” to successfully edit all of the desired changes into the genome.

Another, mechanistically informative error that we detected was complete or partial editing with duplication of sequences corresponding to the template (Figure 5b–e). A clue as to their genesis lies in the fact that linear DNA transformed into fission yeast can undergo end resection and form concatemers [46]. Thus, our data can be explained by a model in which the transformed template DNA forms concatemers prior to being used (just like template monomers) for HR-based repair of the genomic DSB. Further evidence for this model lies in the junctions between template repeats (Figure 5f), which contain sequences diagnostic for microhomology-mediated joining of resected DNA ends [47]. Moreover, as predicted by our model and by the distance-dependent effects described above, the tracks of editing from concatemeric templates were all continuous in that they included all features from the location of the DSB up to a given point, beyond which they contained no features (Figure 5b,e).

Interestingly, for three different HR templates, we identified clones with successfully edited targets that contained additional mutations that were not designed to be present (Figure 5c–e). Possible sources include some level of pre-existing mutations in the synthetic template DNA molecules or de novo mutations arising in the course of the template-dependent DSB repair. The finding that the same bp change occurred in tandem repeats is more consistent with the former mechanism. Be that as it may, the fact that such mutations can arise at all compromises the overall fidelity of genome editing.

Lastly, we wish to emphasize two points. First, *SpEDIT* yielded successfully edited clones for each of the 15 different template DNA molecules. Second, all of the undesired outcomes were either predicted to occur at some non-zero rate based on the mechanisms of CRISPR, or provided new insight into underlying mechanisms, or both. Looking forward, it will be interesting to further define the parameters underlying the distance-dependent decay in the fidelity of editing in fission yeast, and to develop methods that can achieve higher fidelity, template-directed repair over greater distances.

### 4.3. Summary and Implications

The fission yeast-optimized, *SpEDIT* methodology can be used successfully to generate a diverse collection of DNA sequence elements within a target locus of interest. However, the approach can also generate a wide variety of undesired, on-target changes. This new knowledge will help researchers make informed decisions on whether to use CRISPR-based [25,26,27,28,29,30], PIPO-based [21,22], vectorette-based [23,24], or recombinase-based [48,49] approaches for their particular genome editing applications.

## Figures and Tables

**Table 1:** **DNA Oligonucleotides**.

Name	Sequence
ade6sgRNA-F	5′-CTAGAGGTCTCGGACTACAGTTCAACAATTGCATCGGTTTCGAGACCCTTCC-3′
ade6sgRNA-R	5′-GGAAGGGTCTCGAAACCGATGCAATTGTTGAACTGTAGTCCGAGACCTCTAG-3′
HRuniv-R	5′-CGGCTGCCAAGGCATCAGTGTTAATATGCTCAATTTCAGTTGTTAATAACGTGCACTTCTTAGACAGTTCAACAATTGCATCGCCATCAGTAAATGATGC-3′
HES92-F	5′-GCTTAAACATCAAATGCATCATCTTGGATGCAGCAAATTCTCCTGCCTAACGTTACGTTAATTTTGGTGAGCACATTGATGCATCATTTACTGATGGCGA-3′
HES95-F	5′-GCTTAAACATCAAATGCATCATCTTGGATGCAGCAAATTCTCCTGCCTAACGCAAAAGATAGATCGGTGAGCACATTGATGCATCATTTACTGATGGCGA-3′
HES96-F	5′-GCTTAAACATCAAATGCATCATCTTGGATGCAGCAAATTCTCCTGCCTAACTTTGCGGATAAAGCAGTGAGCACATTGATGCATCATTTACTGATGGCGA-3′
HES98-F	5′-GCTTAAACATCAAATGCATCATCTTGGATGCAGCAAATTCTCCTGCCTAACGACGGAAAAACTCTAGTGAGCACATTGATGCATCATTTACTGATGGCGA-3′
HES99-F	5′-GCTTAAACATCAAATGCATCATCTTGGATGCAGCAAATTCTCCTGCCTAACCACTCGTTCTAGCCTGTGAGCACATTGATGCATCATTTACTGATGGCGA-3′
HES102-F	5′-GCTTAAACATCAAATGCATCATCTTGGATGCAGCAAATTCTCCTGCCTAACTCGCTTCGTCATCGCGTGAGCACATTGATGCATCATTTACTGATGGCGA-3′
HES113-F	5′-GCTTAAACATCAAATGCATCATCTTGGATGCAGCAAATTCTCCTGCCTAACGGGTACTATTACCCGGTGAGCACATTGATGCATCATTTACTGATGGCGA-3′
HES120-F	5′-GCTTAAACATCAAATGCATCATCTTGGATGCAGCAAATTCTCCTGCCTAACCAATAAAAGGGCGGGGTGAGCACATTGATGCATCATTTACTGATGGCGA-3′
HES122-F	5′-GCTTAAACATCAAATGCATCATCTTGGATGCAGCAAATTCTCCTGCCTAACAGCCCAGATATTAGGGTGAGCACATTGATGCATCATTTACTGATGGCGA-3′
HES199-F	5′-GCTTAAACATCAAATGCATCATCTTGGATGCAGCAAATTCTCCTGCCTAACCAATCAGAAATAGTCGTGAGCACATTGATGCATCATTTACTGATGGCGA-3′
HES226-F	5′-GCTTAAACATCAAATGCATCATCTTGGATGCAGCAAATTCTCCTGCCTAACGTTTCAAGCCCTCTCGTGAGCACATTGATGCATCATTTACTGATGGCGA-3′
HES231-F	5′-GCTTAAACATCAAATGCATCATCTTGGATGCAGCAAATTCTCCTGCCTAACCAAAGCGACGTAATAGTGAGCACATTGATGCATCATTTACTGATGGCGA-3′
HES253-F	5′-GCTTAAACATCAAATGCATCATCTTGGATGCAGCAAATTCTCCTGCCTAACGTTAGATCAGAAAGCGTGAGCACATTGATGCATCATTTACTGATGGCGA-3′
HES397-F	5′-GCTTAAACATCAAATGCATCATCTTGGATGCAGCAAATTCTCCTGCCTAACAGGGTGGGCGTGTGAGTGAGCACATTGATGCATCATTTACTGATGGCGA-3′
HEScon-F	5′-GCTTAAACATCAAATGCATCATCTTGGATGCAGCAAATTCTCCTGCCTAACAAATTGATGGAGGACGTGAGCACATTGATGCATCATTTACTGATGGCGA-3′
ade6+63-F	5′-GGCAGCCCATCGCTTAAACA-3′
ade6+258-R	5′-CGTAACGGCTGCCAAGGCAT-3′
ade6-57-F	5′-CAACATTTACCATCTCATTAAGCTGAG-3′
ade6+984-R	5′-TGAAACATAATCAGGATCATCAGTACC-3′

## Data Availability

All the data necessary to support the conclusions of this study are contained within the article. Yeast strain reagents are available from the authors upon request.
